# Case Report: Identification of a novel *LYN::LINC01900* transcript with promyelocytic phenotype and *TP53* mutation in acute myeloid leukemia

**DOI:** 10.3389/fonc.2023.1322403

**Published:** 2023-12-01

**Authors:** Chengjun Hu, Qiuxin Dai, Ruiyi Zhang, Huanping Yang, Man Wang, Kaili Gu, Jiangang Yang, Wenjun Meng, Ping Chen, Maozhong Xu

**Affiliations:** ^1^ Department of Hematology, The Affiliated Jiangyin Hospital of Southeast University Medical College, Jiangyin, Jiangsu, China; ^2^ Suzhou Jsuniwell Medical Laboratory, Suzhou, China; ^3^ Jiangsu Institute of Hematology, National Clinical Research Center for Hematologic Diseases, NHC Key Laboratory of Thrombosis and Hemostasis, The First Affiliated Hospital of Soochow University, Suzhou, China

**Keywords:** acute myeloid leukemia, LYN::LINC01900, promyelocytic phenotype, lncRNA fusion, mutation

## Abstract

Acute myeloid leukemia (AML) is a malignant disease of myeloid hematopoietic stem/progenitor cells characterized by the abnormal proliferation of primitive and naive random cells in the bone marrow and peripheral blood. Acute promyelocytic leukemia (APL) is a type (AML-M3) of AML. Most patients with APL have the characteristic chromosomal translocation t(15; 17)(q22; q12), forming *PML::RARA* fusion. The occurrence and progression of AML are often accompanied by the emergence of gene fusions such as *PML::RARA*, *CBFβ::MYH11*, and *RUNX1::RUNX1T1*, among others. Gene fusions are the main molecular biological abnormalities in acute leukemia, and all fusion genes act as crucial oncogenic factors in leukemia. Herein, we report the first case of *LYN::LINC01900* fusion transcript in AML with a promyelocytic phenotype and *TP53* mutation. Further studies should address whether new protein products may result from this fusion, as well as the biological function of these new products in disease occurrence and progression.

## 1 Introduction

Acute myeloid leukemia (AML), the most common acute leukemia (AL) among adults, is a highly heterogeneous hematological malignant tumor characterized by the proliferation and abnormal differentiation of immature cloned myeloid cells (1). Acute promyelocytic leukemia (APL) is a type (AML-M3) of AML. Most patients have a specific chromosomal translocation t(15;17)(q22;q12), forming *PML::RARA* gene fusion, whose protein product leads to cell differentiation arrest and insufficient apoptosis, which is the main molecular mechanism of APL occurrence (2). Initial, prompt diagnosis of APL is made through morphological and flow cytometric analyses. APL typically displays a distinct immunophenotype that lacks HLA-DR, CD34, and CD11B expression (3, 4). However, similar immunophenotypic results have been observed in other AML types (5, 6). Thus, diagnosis of this APL-like AML has been a major challenge, and the lag between onset and diagnosis, as well as the resistance to the routine treatment of classical APL, results in an unfavorable prognosis (7, 8).

Many researchers have found that the onset of AML is usually accompanied by the appearance of different gene fusions such as *CBFB::MYH11*, *PML::RARA* and *RUNX1::RUNX1T1 (* 9–11). Gene fusions are the main molecular markers for prognostic stratification, minimal residual disease (MRD) monitoring, and targeted therapy in patients with AML (12). Furthermore, interactions between gene fusions and mutated genes can play a crucial role in the prognosis and recurrence of AML (13). With an increasing number of new fusions identified, hematopoietic malignancies have been shown to have greater molecular diversity, which may have important implications for sophisticated subtyping with molecular markers.

Long non-coding RNA (lncRNA) is defined as a non-coding RNA molecule with a length exceeding 200 nucleotides that was once considered as transcriptional ‘junk’ DNA (14). However, with the development of molecular biology and sequencing technology, lncRNAs have been found to play an important role in tumor cell growth, apoptosis, invasion, and metastasis, as well as in the occurrence and progression of diseases (15, 16).

Herein, we identified the *LYN::LINC01900* fusion transcript, which was predicted to express no fusion protein in an AML patient with *TP53* mutation. Moreover, clinical evidence showed that the bone marrow cells of the patient retained a promyelocytic phenotype; but there was no *PML::RARA* fusion. This indicated the potential function of this fusion in a new AML subtype. Hence, we hope that this case report on *LYN::LINC01900* transcript will provide a new perspective for understanding the occurrence and progression of AML.

## 2 Case description

A 76-year-old man was admitted to our hospital on October 12, 2022, with dizziness and fatigue for 6 months that had aggravated one month prior. The patient had a history of hypertension for over 20 years and type 2 diabetes mellitus and renal insufficiency for 8 and 2 years, respectively. After admission, a complete routine peripheral blood examination showed a white blood cell count of 1.26*10^9/L, red blood cell count of 1.44*10^12/L, monocyte cell count of 0.05*10^9/L, neutrophil cell count of 0.38*10^9/L, hemoglobin level of 51.0 g/L and platelet count of 32*10^9/L. The lymphocyte count was normal. The fibrinogen and D-dimer levels were 3.30 g/L (reference values [ref.] 2.00–4.00 g/L) and 0.55 mg/L (ref. 0.00–0.55 μg/mL), respectively. The prothrombin and activated partial thromboplastin time were 12.5 s (ref. 10.0–14.0 s) and 27.2 s (ref. 25–31.3 s), respectively.

**Figure 1:**
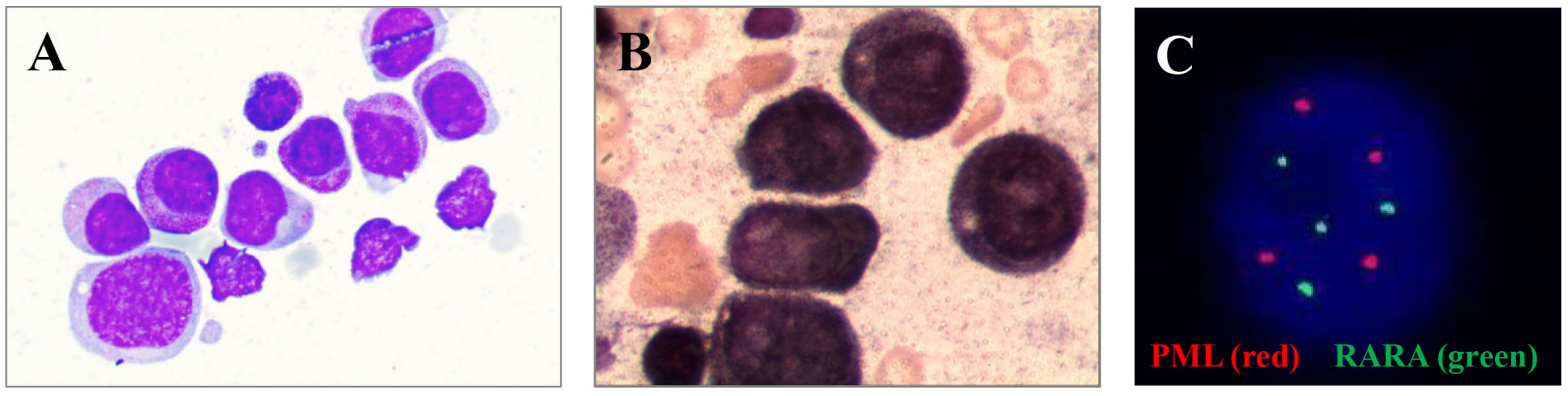
**(A)** Bone marrow smear of primitive granulocytes under a microscope. **(B)** Original granulocyte bone marrow smear after peroxidase staining. **(C)** FISH results of BM cells.

To understand the cytological morphology, we observed the bone marrow smear of the patient and found that bone marrow cells proliferation and granulocyte proliferation were markedly high. The proportion of primitive granulocytes was 28%, and that of promyelocytes was as high as 56.5%. In addition, Wright Giemsa staining showed that erythrocyte and lymphocytic proliferation were inhibited (**Figure 1A**). We further performed peroxidase staining test and found that the results of peroxidase staining of the bone marrow cell smear were positive (**Figure 1B**). Immunophenotyping by flow cytometry showed that the granulocyte population accounted for 58.5%, and these cells were positive for CD117, CD33, and CD38; weakly expressed CD13, CD4, CD64, and CD45; and negative for CD7, CD34, HLA-DR, CD10, CD20, CD19, CD14, CD2, CD15, CD11B, CD56, CD8, and CD3.

**Figure 2:**
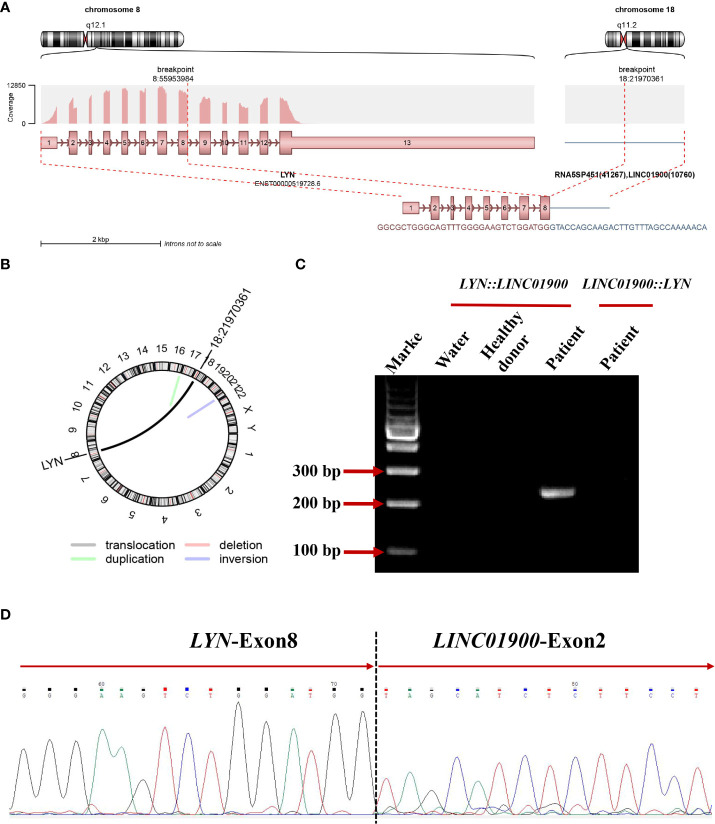
**(A)** Schematic diagram of the formation mechanism of *LYN::LINC01900* transcript. **(B)** Circos plot indicating novel fusions between *LYN* and *LINC01900*. **(C)** Electrophoresis of RT-PCR products from patient showing the *LYN::LINC01900* fusion transcript. **(D)** Partial nucleotide sequences surrounding the junctions of the *LYN::LINC01900* fusion transcript.

Subsequently, the karyotype of the patient was analyzed. The results revealed a complex karyotype of 73–80, XXY, +1, +2, –3, +4, +6,?der (7), +8, –9, –9, –11, –11, –12, +13, +14, +15, +15, +16, ?17, der (17)?i(17q), +19, +19, +20, +21, +22, +mar, inc[CP3]/45, X, –Y[5]/46, XY[2]. FISH showed no evidence of *PML::RARA* fusion invloving t (15, 17)(q24; q21) but showed a higher fluorescence signal (normally 2 red and 2 green in single cell, now 4 red and 4 green), indicating gene amplification or +15,+15,+17,+17, which may originate from aberrant cells (**Figure 1C**). To further identify whether there were molecular variations involving essential genes functioning in hematopoietic malignancies, we used a 45-gene panel to detect gene mutations and a 53-gene panel to conduct a more comprehensive fusion screening using targeted RNA sequencing. Gene mutation results showed that *TP53* p.Arg273Cys was detected with a mutation allele frequency (MAF) of 81.4%. *WT1* p.Thr277Ile was detected, and the *WT1* mutation was suspected to be a germline mutation, based on the MAF of 67.10%. We used STAR software for bioinformatics analysis to predict fusion genes, with the reference genome being hg38. The *LYN::LINC01900* transcript, which is a fusion of *LYN* (NM_001111097.3) exon 8 with *LINC01900* (NC_000018.10) exon 2, was detected at a relative transcript expression level of 15.92% normalized to the reference gene *ABL1* (**Figures 2A, B**). We then performed agarose gel electrophoresis on the amplified products (water as a blank control and healthy donor cDNA as a negative control). A band with a length of 230 bp was detected (100 bp of marker), while the reciprocal fusion *LINC01900::LYN* was not found, consistent with the RNA-targeted sequencing results (**Figure 2C**). Sanger sequencing of the targeted fusion product confirmed the presence of the *LINC01900::LYN* fusion transcript (**Figure 2D**).

**Figure 3:**
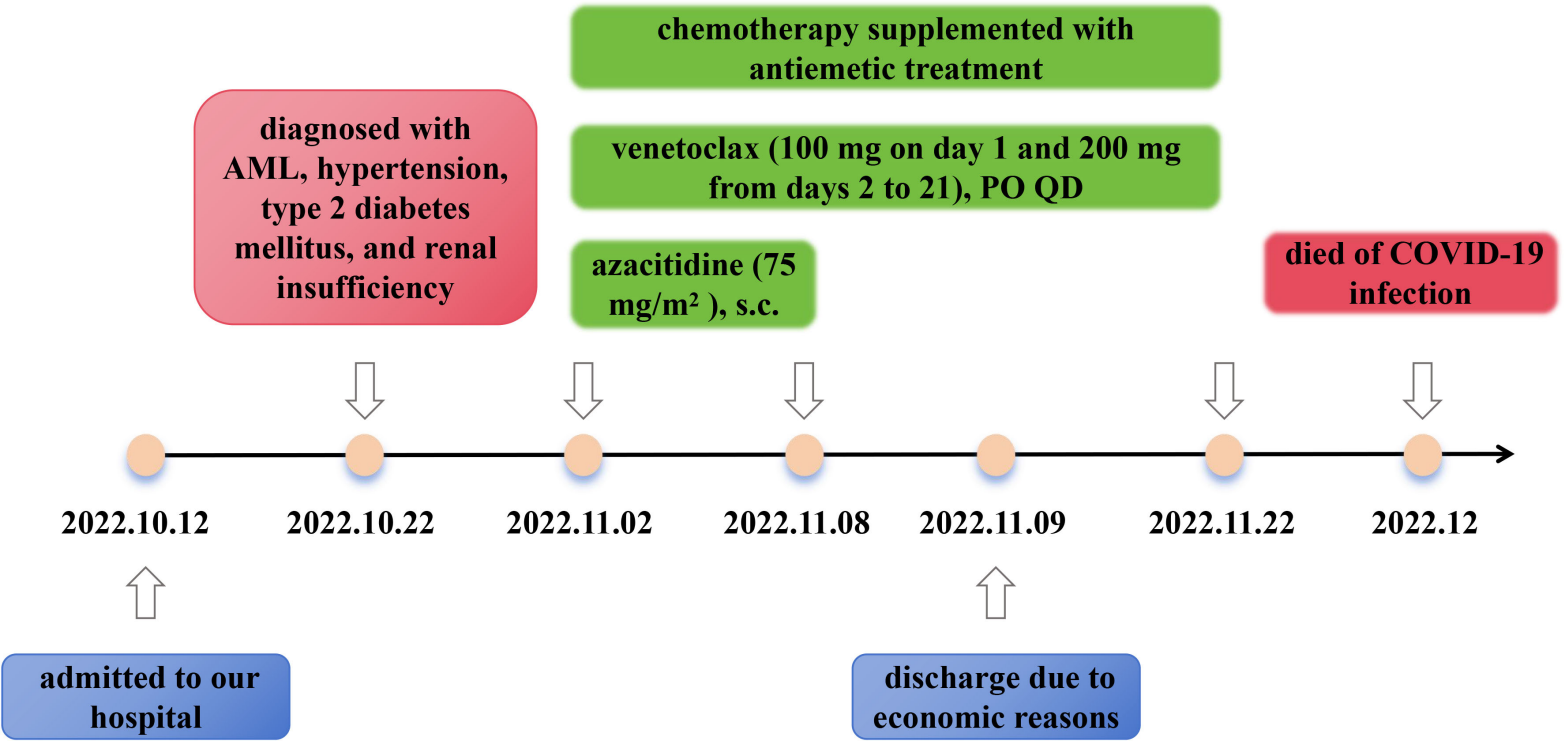
Timeline of the treatment.

The standard azacitidine + venetoclax therapy regimen, which consists of venetoclax (100 mg for day 1, 200 mg for day 2, and 400 mg from days 3 to 28), PO QD, and azacitidine (75 mg/m^2^ from days 1 to 7), subcutaneous injection (s.c.), was recommended according to the 2022 European LeukemiaNet (ELN) recommendations for the diagnosis and management of AML in adults (17). Considering the patient’s renal dysfunction, the treatment was finally adjusted to venetoclax (100 mg on day 1 and 200 mg from days 2 to 21), PO QD, and azacitidine (75 mg/m^2^ from days 1 to 7), s.c., supplemented with antiemetic treatment at the same time from November 02, 2022. The therapy regimen was adopted after communicating with the patient and their family members. The patient discharged due to economic reasons on November 09, 2022. After discharged, we conducted a follow-up finding that the patient continued the therapy of venetoclax (200 mg, PO QD) as we suggested until November 22, 2022. Unfortunately, no further clinical outcomes were monitored because the patient died of COVID-19 infection on December 2022 (**Figure 3**).

## 3 Discussion

*LYN*, which belongs to the *ABL/Src* tyrosine kinase family, is a proto-oncogene expressed in many hematopoietic diseases, including AML (18). The functional domains of *LYN* include four major parts: Src Homology 2 (SH2), SH3, proline-rich hinge region (P), and tyrosine kinase domain (19). The tyrosine kinase encoded by *LYN* is mainly expressed in hematopoietic cells, nervous tissue, liver, and adipose tissue (20). It also plays an important role in B cell-related signaling, mast cell development, and erythrocyte differentiation (21). Currently, gene rearrangements involving *LYN* are rarely observed in hematological diseases. There are a total of 6 reported cases of rearrangement involving *LYN* in hematological diseases, among which *ETV6::LYN* has been reported in 3 cases across different disease types including AML, primary myelofibrosis (PMF), and myeloproliferative neoplasms (MPN) (22–24). The clinical outcomes of the patients after receiving allogeneic hematopoietic stem cell transplantation (allo-HSCT) and/or chemotherapy showed two deaths and one not available. In a study from the children’s oncology group, Shalini et al. reported the first *GATAD2A::LYN* transcript in B-cell acute lymphoblastic leukemia (B-ALL). However, no other relevant clinical data have been found in this paper (25). Yano et al. reported a *NCOR1::LYN* transcript accompanied with additional deletion of *IKZF1*, *CDKN2A/2B*, and *BTG1* in B-ALL (26, 27). Also in B-ALL, Dai et al. found a *NCOR1::LYN* transcript accompanied with deletion of *IKZF1* and *CDKN2A*. After receiving allo-HSCT and chemotherapy, the two patients received complete remission (CR) and complete molecular remission (CMR), respectively (28)(**Table 1**).

**Table 1:** The characteristics of the reported and our case with a *LYN* rearrangement.

Age (yrs)	Gender	Disease	Initial WBC count (x 10^9^/L)	Karyotype	Gene fusion	Additional genetic changes	Therapy	Clinical outcome	Cases
21	male	PMF	25.5	46XY, ins (12,8)(p13;q11q21)	*ETV6::LYN*	not available	Chemotherapy and hllo-HSCT	Dead	H Tanaka et al. (22)
46	male	MPN	17.2	46,XY,der (8)inv(q12.1q21.1)t(8;12)(q12.1;p13),der(12)t(8;12)(q12.1;p13)[2]/47,sl,+der(8)inv(8)t(8;12)[5]/48,sdl1,+der(8)inv(8)t(8;12)[2]/46,XY[2]	*ETV6::LYN*	not available	Chemotherapy	Dead	N Telford et al. (23)
41	male	AML	16.1	47,XY,der(1)ins(1;8)(p13;q12.1q24.21)t(1;12)(p13;p13.2),+8,der(8)ins(1;8)(p13;q12.1q24.21),der(12)t(1;12)(p13;p13.2)[19]/46,XY[1]	*ETV6::LYN*	no	Chemotherapy and hllo-HSCT	not available	Edmond S.K. Ma et al. (24)
not available	not available	B-ALL	not available	not available	*GATAD2A::LYN*	not available	not available	not available	Shalini C. Reshmi et al. (25)
8	female	B-ALL	293	no metaphase	*NCOR1::LYN*	*IKZF1*, *CDKN2A/2B*, *BTG1* deletion	Chemotherapy and hllo-HSCT	CR	Mio Yano et al. (26) and T Imamura et al. (27)
6	male	B-ALL	883	46,XY,t (8,17)(q12;p11.2[10]/48,idem,+der(17)t(9;17),+22/46,XY[1]) [9]/46, XY[9]	*NCOR1::LYN*	*IKZF1*,*CDKN2A* deletion	Chemotherapy and hllo-HSCT	CMR	Hai-Ping Dai et al. (28)
76	male	AML	1.26	73–80, XXY, +1, +2, –3, +4, +6,?der (7), +8, –9, –9, –11, –11, –12, +13, +14, +15, +15, +16, ?17, der (17)?i(17q), +19, +19, +20, +21, +22, +mar, inc[CP3]/45, X, –Y[5]/46, XY	*LYN::LINC01900*	*TP53* mutation	Chemotherapy	Dead	The present case

A new rearrangement of *LYN* was found in this patient. This molecule was an lncRNA named *LINC01900*, lncRNAs have been reported to play a role in tumor cell growth, apoptosis, invasion, and metastasis, as well as in the occurrence and development of diseases by acting as chromatin-modifying factors, X chromosome-inactivating factors, enhancers, transcription regulating factors, and post-transcription regulating factors (29–32). *LYN::LINC01900* is formed through translocation, with *LYN* breaking at chr8:55953984:+ and *LINC01900* breaking at chr18:22043217:+. This transcript was an out-of-frame fusion protein that did not produce chimeric proteins. However, it may produce a truncated or possibly non-produced *LYN* protein, indicating the pathogenic role of haploid *LYN* dysfunction in this patient. In addition, no evidence of the reciprocal fusion transcript *LINC01900::LYN* was found. This indicates a more complicated translocation process that may be coupled with further transcriptional or post-transcriptional regulation.

**Figure 4:**
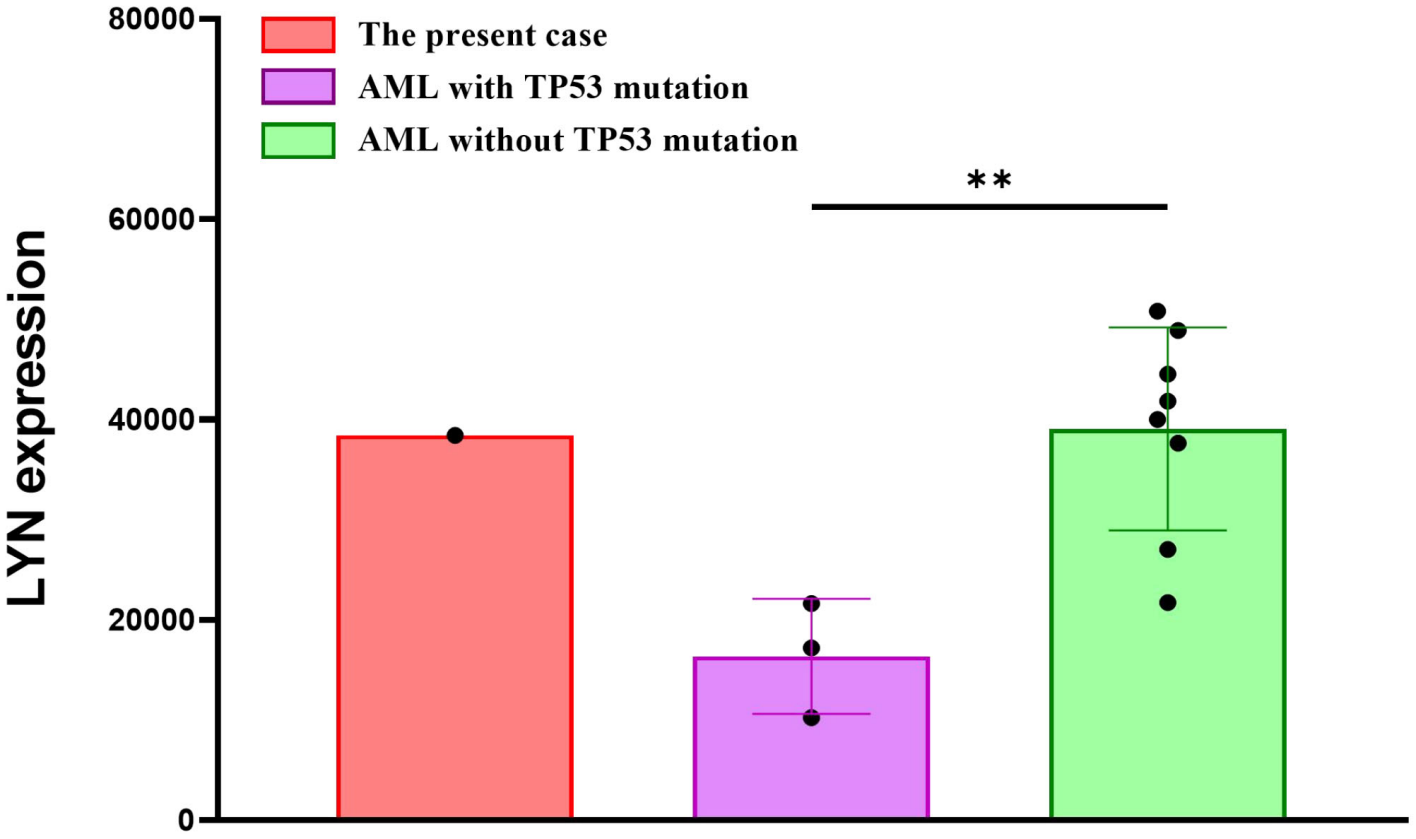
The mRNA expression of *LYN* in the AML patients with/without *TP53* mutation, and the present case.

Most patients with APL have a specific chromosomal translocation, t (15, 17)(q22; q12), forming the *PML::RARA* fusion gene. In our case, the cells retained a promyelocytic phenotype based on blast morphology and flow cytometric analysis. However, the FISH results of *PML::RARA* fusion gene in bone marrow of this patient suggested that there was no sign of t (15, 17)(q24; q21), but there were higher fluorescence signals indicating gene amplification. Studies have reported that APL-like AML is often accompanied by mutations in *NPM1* and/or *FLT3* mutation (33, 34). However, no *NPM1* mutation was detected. We found a *TP53* (NM_000546.5) p.Arg273Cys mutation with a MAF of 81.4%. To the best of our knowledge, this is the first case of APL-like AML with *TP53* mutation, which is the most important tumor suppressor factor and is crucial for maintaining cellular genomic integrity. These findings may be used to identify new AML subtypes. Studies have indicated that AML with *TP53* mutations is often accompanied by complex karyotypes, which is consistent with the findings in our case (35, 36). Interactions between fusion and mutated genes can also play a crucial role in the prognosis and recurrence of AML (13). *LYN::LINC01900* may affect AML progression by interacting with *TP53*. Interestingly, we found that the expression of *LYN* was significantly reduced in AML patients with *TP53* mutation, compared to the AML patients without *TP53* mutation. The transcript per million (TPM) values from RNA-sequencing of the present case appear to be higher than those of the AML patients with *TP53* mutations, indicating that the *LYN* expression in this fusion may increase, which may cause the progression of diseases by regulating expression of other genes related to AML (**Figure 4**). Further clinical data and reports need to be analyzed to improve this study in the future.

Although the new fusion transcript was confirmed using different methods, no significant differences in expression of the fusion partner genes were observed. Moreover, no fusion protein was predicted because there may have been no termination codons. Further studies should address whether new protein products may result from this fusion, as well as the biological function of these new products in disease occurrence and progression.

## 4 Conclusions

Here, we report the case of a 76-year-old man with AML and a significantly higher proportion of promyelocytes. Wright Giemsa compound staining and peroxidase staining showed that erythrocyte and lymphocytic proliferation were inhibited, and the results of peroxidase staining of the bone marrow cell smear were positive. Immunophenotyping showed that the granulocyte population was positive for CD117, CD33, and CD38; weakly expressing CD13, CD4, CD64, and CD45; and negative for CD7, CD34, HLA-DR, CD10, CD20, CD19, CD14, CD2, CD15, CD11B, CD56, CD8, and CD3. Although a complex chromosomal karyotype was identified, FISH, qPCR, and RNA-targeted sequencing confirmed no evidence of *PML::RARA*. Additionally, *TP53* p.Arg273Cys was identified in the present case. Taken together, this patient was diagnosed with AML with *TP53* mutation and a promyelocyte phenotype. A novel fusion *LYN::LINC01900* was identified in this case. However, reciprocal fusion was not observed. Furthermore, the fusion was predicted to not express fusion proteins. Future studies should focus on the subgroups of fusions involving non-coding RNAs and their biological functions in hematopoietic malignancies. In conclusion, the *LYN::LINC01900* fusion transcript from the patient is the first such fusion to be reported on a global scale, to our knowledge. Here, we share this case report a novel *LYN::LINC01900* transcript with *TP53* mutation in APL-like AML to provide a new perspective for understanding the molecular diversity of AML.

## Data availability statement

The datasets presented in this study can be found in online repositories. The names of the repository/repositories and accession number(s) can be found in the article/Supplementary Material.

## Ethics statement

The studies involving humans were approved by Department of Hematology, The Affiliated Jiangyin Hospital of Southeast University Medical College, Jiangyin, Jiangsu, China. The studies were conducted in accordance with the local legislation and institutional requirements. The participants provided their written informed consent to participate in this study. Written informed consent was obtained from the individual(s) for the publication of any potentially identifiable images or data included in this article.

## Author contributions

CH: Data curation, Writing – review & editing. QD: Data curation, Writing – review & editing. RZ: Data curation, Writing – original draft, Writing – review & editing. HY: Data curation, Writing – review & editing. MW: Data curation, Writing – review & editing. KG: Data curation, Writing – review & editing. JY: Data curation, Writing – review & editing. WM: Data curation, Writing – review & editing. PC: Data curation, Methodology, Writing – review & editing. MX: Data curation, Methodology, Writing – review & editing.
